# Lower Rate of Daily Smokers With Symptomatic COVID-19: A Monocentric Self-Report of Smoking Habit Study

**DOI:** 10.3389/fmed.2021.668995

**Published:** 2022-01-05

**Authors:** Makoto Miyara, Florence Tubach, Valérie Pourcher, Capucine Morélot-Panzini, Julie Pernet, Julien Haroche, Said Lebbah, Elise Morawiec, Guy Gorochov, Eric Caumes, Pierre Hausfater, Alain Combes, Thomas Similowski, Zahir Amoura

**Affiliations:** ^1^Sorbonne Université, Inserm UMR-S 1135, Centre d'Immunologie et des Maladies Infectieuses (CIMI-Paris), Groupe Hospitalier Universitaire APHP.Sorbonne-université, site Pitié-Salpêtrière, Département d'immunologie, Paris, France; ^2^Sorbonne Université, Inserm UMR-S 1136, Institut Pierre Louis d'Epidémiologie et de Santé Publique, Groupe Hospitalier Universitaire APHP.Sorbonne-Université, site Pitié-Salpêtrière, Département de Santé Publique, Unité de Recherche Clinique Pitié, CIC-1422, Paris, France; ^3^Sorbonne Université, Inserm UMR-S 1136, Institut Pierre Louis d'Epidémiologie et de Santé Publique, Groupe Hospitalier Universitaire APHP.Sorbonne-Université, site Pitié-Salpêtrière, Service des maladies infectieuses et tropicales, Paris, France; ^4^Sorbonne Université, Inserm, UMRS-1158, APHP, Groupe Hospitalier Universitaire APHP- Sorbonne Université, site Pitié-Salpêtrière, Service de Pneumologie et Réanimation Médicale (Département R3S), Paris, France; ^5^Sorbonne Université, GRC-14 BIOSFAST, UMR Inserm 1166, IHU ICAN, Service d'accueil des Urgences, Groupe Hospitalier Universitaire APHP.Sorbonne-université, site Pitié-Salpêtrière, Paris, France; ^6^Sorbonne Université, Inserm UMR-S 1135, Centre d'Immunologie et des Maladies Infectieuses (CIMI-Paris), Groupe Hospitalier Universitaire APHP.Sorbonne-université, site Pitié-Salpêtrière, service de médecine interne 2, Paris, France; ^7^APHP, Groupe Hospitalier Universitaire APHP.Sorbonne Université, site Pitié-Salpêtrière, Service de Pneumologie et Réanimation Médicale (Département R3S), Paris, France; ^8^Sorbonne Université, Inserm, UMRS_1166-ICAN, Institute of Cardiometabolism and Nutrition, APHP. Sorbonne-université, Service de médecine intensive-réanimation, Institut de Cardiologie, site Pitié-Salpêtrière, Paris, France

**Keywords:** tobacco, SARS-CoV-2, cross sectional, COVID-19, smoking-epidemiology

## Abstract

**Background:** Identification of prognostic factors in COVID-19 remains a global challenge. The role of smoking is still controversial.

**Methods:** PCR-positive in- and outpatients with symptomatic COVID-19 from a large French University hospital were systematically interviewed for their smoking status, use of e-cigarette, and nicotinic substitutes. The rates of daily smokers in in- and outpatients were compared using the same smoking habit questionnaire to those in the 2019 French general population, after standardisation for sex and age.

**Results:** The inpatient group was composed of 340 patients, median age of 66 years: 203 men (59.7%) and 137 women (40.3%), median age of both 66 years, with a rate of 4.1% daily smokers (CI 95% [2.3–6.9]) (5.4% of men and 2.2% of women). The outpatient group was composed of 139 patients, median age of 44 years: 62 men (44.6%, median age of 43 years) and 77 women (55.4%, median age of 44 years). The daily smoker rate was 6.1% (CI 95% [2.7–11.6], 5.1% of men and 6.8% of women). Amongst inpatients, daily smokers represented 2.2 and 3.4% of the 45 dead patients and of the 29 patients transferred to ICU, respectively. The rate of daily smokers was significantly lower in patients with symptomatic COVID-19, as compared to that in the French general population after standardisation by age and sex, with standardised incidence ratios (SIRs) of 0.24 [0.12–0.48] for outpatients and 0.24 [0.14–0.40] for inpatients.

**Conclusions:** Daily smoker rate in patients with symptomatic COVID-19 is lower as compared to the French general population

## Introduction

The COVID-19 pandemic continues to affect socially heterogeneous patient cohorts. As such, identifying relevant risk factors could allow national public health authorities to implement more targeted and efficient measures to control its spread. The role of smoking, in particular, has been implicated with a worse prognosis in patients with COVID-19 (1), although this remains controversial (2).

Amongst patients hospitalised by *severe acute respiratory syndrome coronavirus-2* (SARS-CoV-2), the crude prevalence of active smokers ranges from 1.4 to 12.5% in China (1, 3–10) to 1.3–5.1% in the USA (11, 12). These data early in the pandemic suggested that the prevalence of active smokers amongst inpatients and outpatients with COVID-19 was much lower compared to the general population. However, these data did not take into account key confounders such as age and sex. Additionally, these studies included mostly hospitalised patients in whom the reported rate of active smoking may be indirectly related to their likelihood of having respiratory or cardiovascular comorbidities. Such patients are more likely to be queried about their smoking status and receive appropriate counselling. On the contrary, the smoking prevalence may be underreported for patients who present with a non-smoking-related condition. These patients are less likely both to be asked about their smoking status and to have it accurately recorded in their medical records. Hence, we consider that the link between active smoking and the risk of SARS-CoV-2 infection has yet to be accurately determined.

To study this, we conducted an observational study that compares the rates of daily active smokers in two groups of patients with COVID-19: (1) admitted or inpatients and (2) non-admitted or outpatients. All data were collected using a dedicated *smoking habit* questionnaire (13). We also standardised our data by patient age and gender.

## Materials and Methods

### Study Design

This was a cross-sectional study investigating the smoking status of patients with COVID-19 who were managed either as in- or outpatients. The inpatients had developed severe symptomatic disease whereas the outpatients had the mild form. We determined the patients' active smoking status using the same *smoking habit questionnaire* as that used in the recent French National Survey of Tobacco Consumption 2019 (13). This allowed us to standardise comparison between our cohort and the national population after accounting for age and gender.

All patients with a confirmed diagnosis of COVID-19 *via* PCR at the Pitié-Salpêtrière Hospital in Paris were eligible. We recruited them from two sources: inpatients [those hospitalised in the medical wards of medicine (excluding ICU)] and outpatients (those after the medical consultation deemed as being well enough to isolate at home). The patients in ICU were excluded as their clinical status made detailed interviewing unfeasible. All inpatient data were collected from 23 March to 9 April 2020 whereas all outpatient data were collected from 28 February to 30 March 2020. We also followed up with all inpatients a month later to collect relevant outcome data.

As per the recommendation of our Ethics and Research Committee of Sorbonne University (2020-CER-2020-13), informed consent was waived.

### Study Endpoints and Definitions

We verified the smoking status of patients by specifically asking whether they were active or former smokers (or had never smoked). For the active smokers, we also asked for further details such as daily or occasional consumption and also the number of cigarettes smoked daily. We used the same definition as that of the Annual Survey of Tobacco Consumption in France (Public Health France Smoking Barometer) (13). Daily smokers were defined as individuals reporting daily consumption of cigarettes or other tobacco products (e.g., cigars, cigarillos, pipe, and shisha). Occasional smokers were those who reported infrequent consumption. Our group of former smokers included anyone who had smoked in the past (occasionally or daily) but had been abstaining before their COVID-19 diagnosis. The term “never smoker” defined patients who had never smoked.

In addition, all patients were asked whether they had used any nicotine replacement therapy (NRT, including e-cigarettes). We asked all former smokers about the duration since they had last smoked and asked active smokers whether they had quit since their diagnosis of COVID-19.

Finally, we extracted the following data from the medical records: admission status (in- or outpatient), age, sex, whether they were healthcare workers, and relevant comorbidities (e.g., diabetes, hypertension, obesity, immune deficiency, and COPD). For the inpatients, we also extracted the following outcomes at one month after their clinical presentation: admission status (with or without ICU stay), discharged without any ICU, or death (in ICU or the ward).

All COVID-19 diagnoses were based on a PCR-positive test from a nasopharyngeal swab.

### Smoking Rates in the National Reference Population

The French population was used as a reference to compute the standardised incidence ratio (SIR). The incidence of daily smokers had already been reported by the French National Survey 2019 (Santé Publique France Health Barometer) (13). This is an annual cross-sectional survey performed on a representative sample of French metropolitan area residents (age range of 18–85) based on a two-stage random sample (13). This survey involved 10, 352 residents and the same definitions of daily smokers, occasional smokers, former smokers, and never smokers as detailed above. The age and gender were reported only for 18–75-year-old active daily smokers but not for occasional active smokers, former smokers, or non-smokers. The rate of active daily smokers in the 76–85-year-old group was reported globally and not by gender.

### Statistical Analyses

A descriptive analysis was performed within each patient group. The qualitative variables were described as frequencies and percentages whereas the quantitative variables were described as median and interquartile range. We accounted for any differences in age and gender between the patient groups *via* Wilcoxon's rank-sum test and chi-squared test. We accounted for any significant differences in comorbidities and smoking status *via* logistic regression (adjusted by age and gender) instead.

The SIRs were used to compare daily smoker rates between the inpatient and outpatient groups, respectively, with those of the reference population. We also separately estimated the SIR in healthcare workers and non-healthcare workers seen in outpatients (as healthcare workers were overrepresented). To estimate SIR and its 95% confidence interval in each group, we used a Poisson regression model with log link and reference rate as offset. Finally, to compare the SIRs between the two groups, we introduced the group variable in the model.

All patients were included in the main analysis. Those older than 75 were analysed as part of the 65–75-year-old group for standardisation (considering the reference rates of daily smokers were 10.4% in men and 9% in women). In our view, this is a conservative approach as the rate of daily smokers decreases with age (only 4.8% of daily smokers amongst the 76–85-year-old French cohort in 2019). As we were unable to confirm the smoking status in six outpatients and two inpatients, we did not include them in the main analysis. Overall, we performed two sensitivity analyses: (1) after excluding patients older than 75 and (2) considering those patients with missing smoking status as daily smokers.

All analyses were performed at a two-sided α level of 5%, using R software, version 3.5.1 (R Foundation for Statistical Computing, Vienna, Austria. URL https://www.R-project.org/).

## Results

### Patient Demographics and Clinical Characteristics

**Figure 1:**
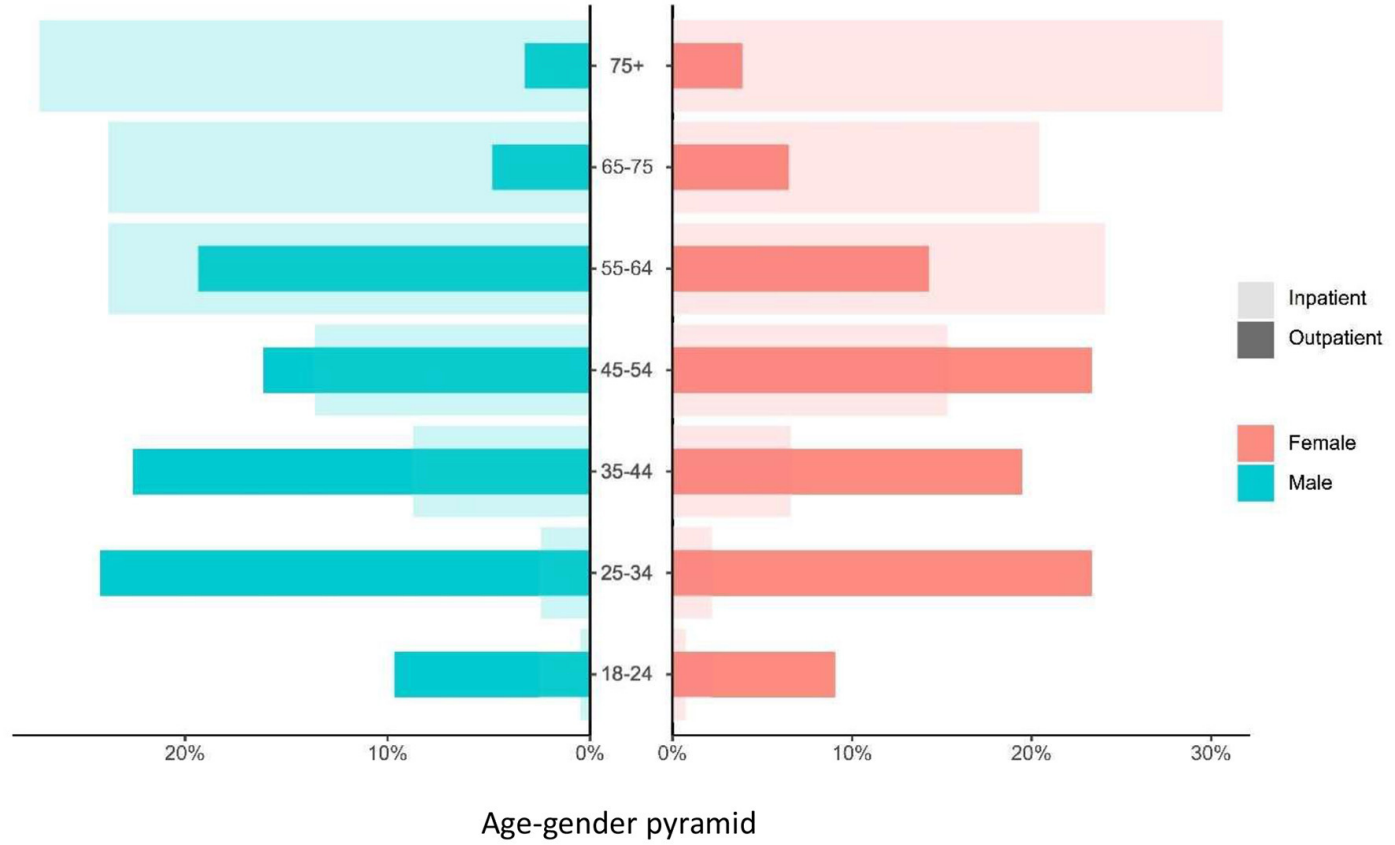
Age and sex distribution in inpatients and outpatients with COVID-19. Dark- and light-shaded histograms represent nonadmitted (outpatients) and admitted (inpatients) patients with confirmed COVID-19 status, respectively. Red represents female patients and blue represents male patients.

The demographic and clinical characteristics of the two groups are shown in Table 1. Overall, we included 340 inpatients and 139 outpatients. The outpatients' cohort was younger than the inpatient cohort (median age of 44 vs. 66, respectively) (Figure 1). Their gender distributions were very different too. In the inpatient group, 59.7% were men compared to 40.3% women whereas, in the outpatient group, 44.6% were men compared to 55.4% women.

**Table 1:** Clinical characteristics and smoking habits of patients with COVID-19.

	**Outpatients (*****N*** **=** **139)**			**Inpatients (*****N*** **=** **340)**			Outpatient/inpatient comparison *p*-value*
	**Male (*n* = 62)**	**Female (*n* = 77)**	**All**	**Male (*n* = 203)**	**Female (*n* =137)**	**All**	
Median (IQR) age (yr)	43 [32–55]	44 [32–54]	44 [32–55]	66 [55–76]	66 [56–79]	66 [55–77]	<0.001
Coexisting disorder
High blood pressure	9 (15.3 %)	7 (9.6 %)	16 (12.1 %)	84 (41.4 %)	58 (42.3 %)	142 (41.8 %)	0.004
Diabetes	4 (6.8 %)	3 (4.1 %)	7 (5.3 %)	54 (26.6 %)	41 (29.9 %)	95 (27.9 %)	<0.001
Obesity	4 (6.78 %)	6 (8.2 %)	10 (7.6 %)	28 (14.3 %)	19 (14.1 %)	47 (14.2 %)	0.003
Immune deficiency	4 (6.8 %)	1 (1.4 %)	4 (3 %)	34 (16.7 %)	26 (19 %)	60 (17.6 %)	<0.001
COPD	2 (3.4 %)	0 (0 %)	2 (1.5 %)	17 (8.4 %)	10 (7.3 %)	27 (7.9 %)	0.381
Smoking status							0.38
Active	3 (5.1 %)	5 (6.8 %)	8 (6.1 %)	11 (5.4 %)	3 (2.2 %)	14 (4.1 %)	
Active occasional	3 (5.1 %)	3 (4.1 %)	6 (4.5 %)	4 (2 %)	0 (0 %)	4 (1.2 %)	
Former	21 (35.6 %)	20 (27.4 %)	41 (31.1 %)	76 (37.6 %)	35 (25.7 %)	111 (32.8 %)	
Never smoker	32 (54.2 %)	45 (61.6 %)	77 (58.3 %)	111 (55 %)	98 (72.1 %)	209 (61.8 %)	
Missing data	4 (6.5 %)	3 (3.9 %)	7 (5.0 %)	1 (0.5 %)	1 (0.7 %)	2 (0.6 %)	

* *Except for age, p-value corresponds to logistic regression models adjusted on age and sex*.

The inpatient group was composed of 203 men (59.7%, median age of 66) and 137 women (40.3%, median age of 66). Fourteen patients in this group were identified as daily smokers. This equated to a rate of 4.1% [CI 95%: 2.3–6.9] with 5.4% being men and 2.2% being women. Amongst them, four smoked five or fewer cigarettes daily, three smoked six to 10 cigarettes, one smoked 15 cigarettes, and five smoked 20 or more. We did not have the data for one patient in this group.

With regard to the former smokers in the inpatient cohort (*n* = 111, 32.8%), we had information on the abstinence period for all except six. Five former smokers (4.8%) had been abstinent for 2 months, 2 (1.9%) for 6 months, and 98 (93.3%) for more than a year before being infected with COVID-19. Two former smokers (1.9%) were using nicotine substitutes (one with e-cigarettes and one with patches) at the time of disease onset.

The outpatient group was composed of 62 men (44.6%, median age of 43) and 77 women (55.4%, median age of 44). Sixty-eight (51.5%) in this group were healthcare workers. Smoking status was missing for seven patients. Eight patients were identified as daily smokers. This equated to a rate of 6.1% [CI 95%: 2.7–11.6] (5.1% of men and 6.8% of women). Amongst them, three smoked <5 cigarettes daily, three smoked 6–10, and two smoked 20 cigarettes or more. Since their diagnosis of COVID-19, two stopped smoking completely without NRT.

With regard to the former smokers in the outpatient cohort (*n* = 41, 31.1%), two (4.9%) had been abstinent for 3 months and 39 (95.1%) for more than a year before being infected with COVID-19. Two (4.9%) were using nicotinic substitutes of which one used e-cigarettes. Finally, amongst the 77 non-smokers, none were using a nicotinic substitute.

Unsurprisingly, the inpatient group was also more multimorbid than the outpatient group. Examples of contributing conditions (after age and gender adjustment) included the following:

- hypertension [*OR*_adj_ = 2.5 (95% CI; 1.4–4.8), *p* = 0.004]
- diabetes [*OR*_adj_ = 5.4 (95% CI; 2.4–13.7) *p* <0.001]
- obesity [*OR*_adj_ = 3.7 (95% CI; 1.7–8.9), *p* = 0.002]
- immune deficiency [*OR*_adj_ = 12.45 (95% CI; 4.6–44.3), *p* < 0.001].

The odds ratio of COPD was not significantly different; *OR*_adj_ = 2.0, *p* = 0.38.

### Comparing the Daily Smoker Rate With the French Population

**Figure 2:**
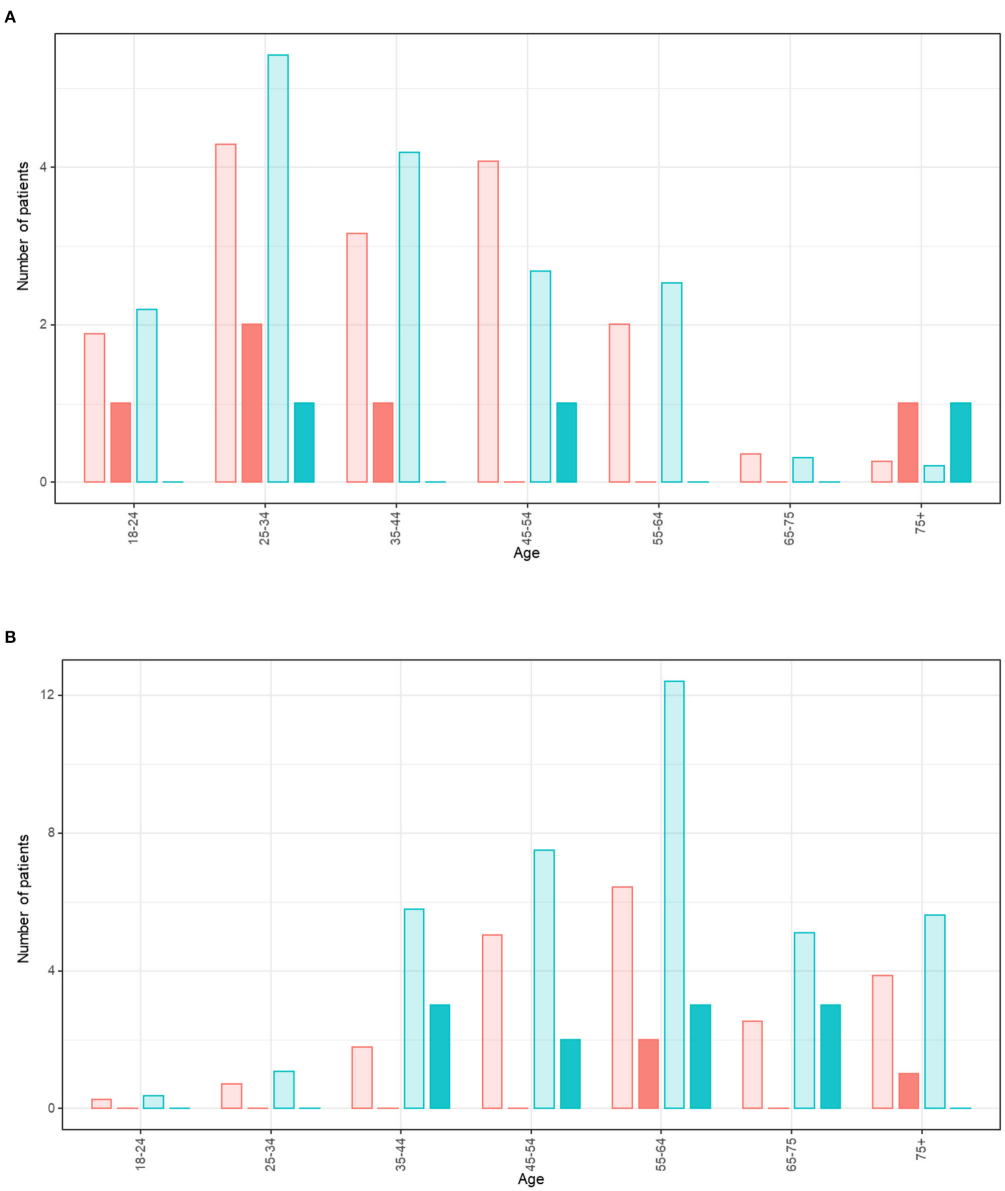
Expected and observed number of cases of daily smokers amongst patients with COVID-19 (categorised by age and gender). **(A)** For outpatients. **(B)** For inpatients. The red bars represent female smokers and blue bars represent male smokers. The bars with lighter shading represent the expected number of daily smokers of each age and gender amongst the patients with COVID-19 in reference to 2019 French general population. The dark bars represent the observed number of daily smokers of each age and gender amongst the patients with COVID-19.

In the main analysis (Figure 2), age- and gender-adjusted SIRs of daily smokers were 0.24 [0.12–0.48] and 0.24 [0.14–0.40] for outpatients and inpatients, respectively (Table 2). Within the outpatients' group, the SIR was 0.17 [0.05–0.53] for the healthcare workers subgroup and 0.32 [0.13–0.76] for the others. Our sensitivity analyses also yielded similar results (Table 2).

**Table 2:** Standardised incidence ratios for daily smokers.

	**SIR CI 95%**	***p*-value**
Main analysis—Inpatients	0.24 [0.14–0.40]	<0.001
Main analysis—Outpatients	0.24 [0.12–0.48]	<0.001
Sensitivity analysis excluding patients older than 75—Inpatients	0.27 [0.15–0.46]	<0.001
Sensitivity analysis excluding patients older than 75—Outpatients	0.18 [0.08–0.40]	<0.001
Sensitivity analysis considering the patients with missing smoking status as daily smokers—Inpatients	0.27 [0.17–0.44]	<0.001
Sensitivity analysis considering the patients with missing smoking status as daily smokers—Outpatients	0.43 [0.26–0.71]	<0.001
Outpatient healthcare workers	0.17 [0.05–0.53]	<0.001
Outpatients without healthcare workers	0.32 [0.13–0.76]	<0.001

*Main analysis involved all included patients. Patients older than 75 were analysed in the 65–75 years of age range for standardisation*.

Of note, the daily smoker rate within the 76–85-year-old patients was 1.6% (inpatients) and 3.8% (outpatients). This was lower than the 4.8% observed in the corresponding age-specific French population (2019 data).

### Outcome of Inpatients With COVID-19

We followed up with all patients in this cohort one-month post-presentation (regardless of active admission status) (Table 3). Fifty-four (15.9%) were still on the medical ward whereas 29 (8.5%) had been transferred to ICU. There had been 46 deaths (13.5%) in ICU or the ward. Finally, 211 (62.1%) had been discharged without requiring any ICU stay.

**Table 3:** Outcomes of patients.

	***n***	**Discharged**	**Still hospitalised**	**Transferred to ICU**	**Died**
Daily smokers	14	9 (64.3%)	4 (28.6%)	1 (7.1%)	1 (7.1%)
Occasional	4	4 (100.0%)	0 (0%)	0 (0%)	0 (0%)
Former smokers	111	58 (52.3%)	19 (17.1%)	11 (9.9%)	23 (20.7%)
Nonsmoker	209	141 (67.5%)	30 (14.4%)	17 (8.1%)	21 (10.0%)
Smoking status unknown	2	0 (0%)	1 (50.0%)	0 (0%)	1 (50.0%)
Total	340	211	54	29	46

*The following outcomes at 1 month after clinical presentation were identified: discharged without any ICU stay, ongoing hospitalisation in the medical ward without ICU admission, or if they occurred earlier: transferred to ICU and still alive at day 30 and finally death (in ICU or the medical ward)*.

Amongst the 14 daily smokers, all were discharged except for one who was transferred to ICU and one who died. Twenty-three former smokers (20.7%) and 21 non-smokers (10%) died whist 11 former smokers (9.9%) and 17 non-smokers (8.1%) were transferred to ICU. Thus, active smokers represented 2.2 and 3.4% of the 45 deaths and the 29 patients transferred to ICU, respectively.

## Discussion

Our monocentric study shows that the rate of daily smokers is significantly lower amongst the patients with symptomatic COVID-19 compared to the French population. This was regardless of the patients' admission status. The SIRs of daily smokers in the outpatients and inpatients groups were identical at 0.24 [0.12–0.48] and 0.24 [0.14–0.40], respectively, which is 76% lower than that of the French population (after adjusting for age and gender).

However, the SIRs did not differ between outpatients and inpatients, suggesting that the potential role of smoking in modulating COVID-19 is independent of the infection severity. We also did not identify a link between infection severity and the number of cigarettes consumed daily. As per the 2019 national data, the mean number of cigarettes smoked daily was 12.5 (13.5 for men and 11.4 for women) (13). Moreover, we also found that nicotinic substitutes had been rarely used by former smokers and never by non-smokers. These findings were in line with our national data indicating that e-cigarette use was low in France overall (4.4% daily users) and that they were very rarely used by non-smokers (1% of e-cigarette users).

Previous studies have also reported a low rate of active smokers amongst patients with COVID-19. In China, this was 1.4–12.6% (1, 3–10) (compared to 27.3% of all adult smokers nationally) whereas, in the USA, this was 1.3% nationally (CDC data) (compared to 14% of all adult smokers nationally). In New York City, this rose to 5.1% instead (11, 12).

Our study collectively investigated the smoking status of outpatients and inpatients infected with COVID-19. Hence, at the time of the study, it was not possible to accurately assess whether the severity of COVID-19 infection was related to active smoking. Patients with severe COVID-19 are generally more multimorbid and may have been previously advised to quit smoking. In the initial data from China, the smoking status of both inpatients and outpatients was not considered separately (1, 3–10). The Centers for Disease Control and Preventions (CDC, USA) found the incidence of active smokers to be 1.3% for their national cohort of patients with COVID-19. More specifically, this was 1% for outpatients, 2% for patients hospitalised but not in an ICU, and 1% for patients admitted to ICU (14). However, it is important to consider that many patients overall did not have their smoking status even recorded in their medical records. Moreover, all other previous studies (except two) have only reported the crude rates of active smokers and not included a control group or the corresponding national population. In those two studies that did include a reference national population, there was neither any statistical comparison nor adjustment for age or gender distribution (1, 14).

Our findings are confirmed by those of other international cohorts. For example, in one Italian study involving patients with COVID-19 admitted to medical wards only, the proportion of active smokers was significantly lower in the COVID-19 group compared to the non-COVID-19 group (4.1% vs. 16%, *p* = 0.00003). Active smokers were also significantly less likely to be hospitalised for COVID-19 compared with non-smokers after adjusting for age and gender (*OR* 0.14; 95% CI, 0.06–0.31, *p* < 0.001) (15). Moreover, in a prospective cohort study using routinely collected data from 1,205 general practitioners in England with 8.28 million participants aged 20–99 years, the proportion of light, moderate, and heavy smokers was also significantly lower in the 19,486 patients who had COVID-19 compared to the total population (5.66 vs. 13.4%, 0.8 vs. 2.58%, and 0.5 vs. 1.19%, respectively) (16, 17). A cross-sectional study in the UK, analysing the smoking status of 3,802 patients registered with the Royal College of General Practitioners Research and Surveillance Centre primary care sentinel network, also found a lower rate of COVID-19 positivity amongst active smokers (11.4%) compared to non-smokers (17.9%) (18). Similar findings were also identified amongst individuals living in homeless shelters in Chicago (19).

Domestically in France, such findings have also been replicated in other regions. In one study (*n* = 661), the active smokers had a lower risk of confirmed COVID-19 compared to non-smokers (7.2 vs. 28.0%; age-adjusted *OR* = 0.23; 95% CI = 0.09–0.59). This association remained significant after adjustment for occupation too (20). Similarly, during the COVID-19 breakthrough that occurred on the Charles de Gaulle aircraft carrier between 21 January to 13 April 2020, the rate of active smokers was lower amongst the COVID-19 infected crewmembers compared to their non-infected colleagues (45 vs. 58%). As per the univariable analysis, this equated to an odds ratio of 0.59 (95% CI; 0.45–0.78; *p* < 0.001) for active smokers vs. former or non-smokers (21). Another study covering the clinical characteristics and factors associated with hospital admission or death in 43,103 adult outpatients described a lower rate of worsening amongst patients who reported being current smokers. The current tobacco use odds ratio was 0.67 [0.47–0.95] for clinical worsening association (22).

Overall, our study has multiple strengths. In contrast to reported work, our study was specifically designed to assess smoking habits in patients with COVID-19. Early studies discussed above had assessed patient smoking status depending on what was recorded in the medical files (1, 3–10). This aspect is often underreported by most clinicians except those involved in respiratory or cardiovascular medicine. We systematically asked patients about their smoking habits and the use of nicotinic substitutes. Although we conducted this study with a systematic and standardised investigation of smoking habits and the use of nicotinic substitutes, in the French context of care, where the smoking status does not impact the access to the best level of care, we cannot completely exclude that self-report in smoking habits might be underestimated and underreported in a context of emergency crisis, but this is unlikely. Moreover, our rate of missing data, one of the most frequent caveats of studies reported so far, was very low (1.9%). Additionally, to completely rule out the impact of missing data on the conclusion of our study, we did a sensitivity analysis that considered patients with missing smoking status as daily smokers. In this analysis, the SIR remained significantly below, thus demonstrating the robustness of our results. Furthermore, we calculated this using the same definitions as those within the French Annual National Survey of Smoking (Public Health France Barometer) (13). Finally, we investigated apart from the association of daily smoking with COVID-19 separately in outpatients and inpatients, which provides additional relevant information to previous studies.

Our study has also certain limitations. First, this work was performed in early 2020 whereas our data on the national reference population dated from 2019. Whilst the difference between both years is likely minimal, we know from previous data that the rate of daily smokers in France has declined in recent years (from 26.9% in 2017 to 24.0% in 2019). In addition, our work looked into the patient population at one hospital and could not be representative of the general population. Our SIRs were calculated based on the assumption that our cohort who mainly originated from the catchment area around a Parisian hospital had the same smoking habits as the general French population. This is important to consider as the rates of smoking rates are lower in the Paris region (22.1% in 2017) compared to other French regions (26.9% in France overall in 2017) (23).

Furthermore, healthcare workers were overrepresented in our outpatient group due to the wider availability of testing at their workplace. Healthcare workers represent a heterogeneous population with similarly heterogeneous rates of smoking habits in France (24) and elsewhere. In a systematic review and meta-analysis, the prevalence of tobacco use in healthcare workers was 21% (31% in men and 17% in women) (25). Additionally, even when estimating the SIR separately in healthcare and non-healthcare outpatients, we still observed significantly lower daily smokers rates in the outpatients than in the general population. Notably, this difference was not identified within our inpatients' cohort where healthcare workers were not overrepresented. It is thus very unlikely that the very low SIRs that were estimated both for the out- and inpatient groups are the result of the study setting (we observed a 76% decrease in the COVID-19 population as compared to the French population, which is very substantial). Smoking rates may differ across ethnic, social status, and socio-professional categories. However, those information were not available in the French national Baromètre Santé survey, preventing us from standardising on these variables. Finally, due to the lack of separately available age or gender data, we were unable to calculate the adjusted SIRs of other subgroups such as former smokers or non-smokers.

A further issue with our study is that we could not include patients admitted to ICU. Hence, we were not able to conclude whether active smoking was associated with very severe forms of COVID-19. Importantly, in our study, active smokers represented 2.2% of the patients who died and 3.4% of those transferred to ICU, respectively. This compares favourably with the rate of 4.1% active smokers in the inpatient cohort. This was also replicated in a multicentre cohort study of 4,244 ICU patients in France, in which the rate of active smokers rate was very low (4%) amongst ICU patients with COVID-19 (26). In addition, Hippisley-Cox et al. also showed a low rate of smokers amongst patients admitted to ICU compared to the total population (3.65 vs. 13.4%, 0.54 vs. 2.58%, and 0.16 vs. 1.19%, respectively) (16).

A further potential issue with our methodology is that the information gleaned was self-reported by patients. This is important to consider in the light of established literature on social desirability bias, which suggests that patients underestimate their real cigarette consumption (27). Although we used the same methodology as the national smoking survey, we consider that any potential bias would have equally affected our study cohort and the national reference cohort in a similar manner. Moreover, as access to healthcare in France is not based upon any private insurance-based health incentive or otherwise, there was no patient advantage in underreporting their smoking status.

In addition, the smoking status in our study was only assessed in patients with symptomatic COVID-19 whereas a proportion of infected individuals can remain asymptomatic (28). Thus, we cannot conclude whether daily smoking is associated with SARS-CoV-2 infection, or with symptomatic forms of this infection. The recent study by Fontanet et al. (20) that relied on SARS-CoV-2 serologies and thus considered both symptomatic and asymptomatic COVID-19 highlighted a decrease in the risk of COVID-19 of the same order of magnitude and provides an answer to this question.

Finally, although our study provides an important perspective in COVID-19 care, our findings remain observational. All things considered, our data may suggest that the effect of tobacco smoking on COVID-19 could be mediated by nicotine rather than whole tobacco smoke. Nicotine can modulate the angiotensin converting enzyme 2 (ACE2) receptor (29–31), which SARS-CoV-2 uses for cellular entry (32–34). This in turn modulates the nicotinic acetylcholine receptor (35). Hence, we hypothesise that SARS-CoV-2 alters the control of the nicotine receptor through acetylcholine. This would explain why previous studies also identified an association between smoking and COVID-19 severity (1, 3, 6). As hospitals generally impose smoking cessation and nicotine withdrawal at the time of hospitalisation, tobacco (nicotine) cessation could lead to the release of nicotine receptors, whose expression is already upregulated in smokers. This could propagate a “rebound effect” responsible for the worsening of disease observed in hospitalised smokers. However, this hypothesis needs further investigation.

The conclusions of our study should be handled with caution. In the light of the possible increased risk of the severe form of COVID-19 amongst smokers once infected and of the long-term harmful consequences of smoking, which is responsible for a very heavy public health burden with more than 78,000 deaths per year in France, our findings need careful consideration and cannot be translated into a clinical practice despite recent studies supporting our conclusions. We want to reaffirm here the deleterious effects of tobacco.

## Data Availability Statement

The raw data supporting the conclusions of this article will be made available by the authors, without undue reservation.

## Ethics Statement

The studies involving human participants were reviewed and approved by Sorbonne University Comité Ethique et Recheche (2020—CER-2020-13). Written informed consent for participation was not required for this study in accordance with the national legislation and the institutional requirements.

## Author Contributions

MM, FT, SL, and ZA designed the study, analysed the data, and wrote the manuscript. VP, CM-P, JP, JH, EM, GG, EC, PH, AC, TS, and ZA recruited the patients, analysed and reviewed the data, and edited the manuscript. All authors contributed to the article and approved the submitted version.

## Conflict of Interest

The authors declare that the research was conducted in the absence of any commercial or financial relationships that could be construed as a potential conflict of interest.

## Publisher's Note

All claims expressed in this article are solely those of the authors and do not necessarily represent those of their affiliated organizations, or those of the publisher, the editors and the reviewers. Any product that may be evaluated in this article, or claim that may be made by its manufacturer, is not guaranteed or endorsed by the publisher.
